# Hybrid selection based multi/many-objective evolutionary algorithm

**DOI:** 10.1038/s41598-022-10997-0

**Published:** 2022-04-27

**Authors:** Saykat Dutta, Rammohan Mallipeddi, Kedar Nath Das

**Affiliations:** 1 Department of Mathematics, National Institute of Technology Silchar, Silchar, India; 2 Department of Artificial Intelligence, School of Electronics Engineering, Kyungpook National University, Daegu, South Korea

**Keywords:** Engineering, Mathematics and computing

## Abstract

In the last decade, numerous multi/many-objective evolutionary algorithms (MOEAs) have been proposed to handle multi/many-objective problems (MOPs) with challenges such as discontinuous Pareto Front (PF), degenerate PF, etc. MOEAs in the literature can be broadly divided into three categories based on the selection strategy employed such as dominance, decomposition, and indicator-based MOEAs. Each category of MOEAs have their advantages and disadvantages when solving MOPs with diverse characteristics. In this work, we propose a Hybrid Selection based MOEA, referred to as HS-MOEA, which is a simple yet effective hybridization of dominance, decomposition and indicator-based concepts. In other words, we propose a new environmental selection strategy where the Pareto-dominance, reference vectors and an indicator are combined to effectively balance the diversity and convergence properties of MOEA during the evolution. The superior performance of HS-MOEA compared to the state-of-the-art MOEAs is demonstrated through experimental simulations on DTLZ and WFG test suites with up to 10 objectives.

## Introduction

Multi-objective optimization problems (MOPs) contain two or more conflicting objectives that need to be optimized simultaneously. MOPs with more than three objectives are referred to as many-objective optimization problems (MaOPs). Due to the conflicting nature of the objectives, the optimization of MOPs results in a set of trade-off solutions instead of a single optimal solution. However, for better decision-making, the trade-off solutions are expected to be optimal and well-spread to cover the entire decision range. Multi/Many-objective Optimization Evolutionary Algorithms (MOEAs), due to their population-based nature, can provide the entire set of trade-off solutions in a single run and are extensively used to solve MOPs. MOEAs try to achieve this with the help of three leading operators, namely mating selection, recombination, and environmental selection. Mating selection is responsible for selecting high-quality parents that form a mating pool and are employed to generate offspring solutions. Recombination process generates quality offspring solutions by combining various solutions (crossover) and/or through perturbation of a single solution (mutation) from the mating pool. Finally, the goal of environmental selection is to select prospective solutions from the combination of parent and offspring solutions for further evolution. As the performance of MOEAs significantly depends on environmental selection, various environmental selection operators have been proposed depending on which MOEAs are classified as Pareto-based^1^, Decomposition^2,3^ or Reference Vector-based, and Indicator-based MOEAs^4^. Each class of MOEAs possesses its advantages and disadvantages in achieving a proper balance between convergence and diversity as the number of objectives increases (i.e., MaOPs) and characteristics of the problem.

Since the output of MOEA is a set of Pareto optimal solutions, the Pareto dominance relation naturally becomes a criterion to distinguish solutions during the evolutionary process. In Pareto-based MOEAs (PMOEAs)^5–8^, when the solutions are not distinguishable due to Pareto dominance, then a diversity criterion is employed where diverse solutions are preferred overcrowded ones. In MaOPs, as the number of objectives increases, the proportion of non-dominated solutions in a population tends to increase exponentially^9^, resulting in the failure of Pareto dominance relation-based criterion in distinguishing solutions. This places more emphasis on density-based secondary selection criterion referred to as ‘active diversity promotion’^10^. The ineffectiveness of Pareto dominance in providing the required selection pressure as the number of objectives increases limits the scalability of PMOEAs. In addition, as the number of objectives increases, the diversity preserving mechanisms such as crowding distance and clustering become computationally expensive. Therefore, reference vectors are employed to reduce the computational complexity and hence the diversity^11^.

Unlike PMOEAs that compare individuals using two criteria (i.e., dominance relation and density), Indicator-based MOEAs (IMOEAs)^12^ adopt a single value referred to as an indicator to measure both convergence and diversity (IBEA^12^, I_SDE_^+^^13^). However, developing an indicator that balances both diversity and convergence is challenging. The ability of the indicator to balance the convergence and diversity gets challenging as the number of objectives increases. Some indicators^12^ are biased towards convergence, while some favour diversity^14^. Therefore, in ^14^, a stochastic combination of both convergence- and diversity-biased indicators are considered. IMOEA based on hypervolume^15^ is effective but computationally expensive. I_SDE_^+^ is computationally efficient but fails to preserve the essential corner solutions.

In Reference Vector-based MOEAs (RV-MOEAs)^16,17^, the population members are guided towards the optimal Pareto Front (PF) in the direction specified by the weight or reference vectors. In general, the reference vectors are selected by sampling a uniform set of points on a hyperplane $\sum_{i}{f}_{i}=1$ in the normalized *M*-objective space referred to as Normal Boundary Intersection (NBI) method^18^. In other words, it is implicitly assumed that the optimal PF is bounded by a unit simplex of reference vectors that is non-degenerate, continuous, and smooth without significant nonlinearities. However, there exist several MOPs characterized by degenerate and discontinuous PFs. As a result, several of the uniform weight vectors fail to get associated with any of the solutions and are referred to as ineffective weight vectors. In addition, it has been observed that the number of non-dominated solutions obtained by MOEA/D^19^, a primitive RV-MOEA, is often much smaller than the number of weight vectors as—(1) multiple weight vectors can share a single good solution, and (2) all solutions are not always non-dominated. Therefore, the Pareto dominance criterion has been integrated into RV-MOEAs (NSGA-III^11,20^, RVEA^21^, MOEA/DD^22^, TDEA^8^, PMEA^23^). On the other hand, a set of uniform weight vectors may not be able to approximate the different sizes and shapes of PFs. In other words, the initialization of the weight vectors should depend on the shape and size of the PF, which may not be known in advance. Therefore, RV-MOEAs^24–27^ with weight vector adaptation during the evolution were proposed to effectively handle MOPs with regular as well as irregular PFs. Instead of adapting the weight vectors, a combination of uniform weight vectors and a secondary criterion (e.g., polar-metric^23^) in to select the solutions corresponding to the ineffective weight vectors have also been investigated. Motivated by the work in^23^, we propose a hybridized framework, referred to as HS-MOEA, that employs I_SDE_^+^ as the secondary criterion to select solutions corresponding to the ineffective weight vectors, in addition to the Pareto dominance. In other words, the aim of this study is to develop a new environmental selection strategy that benefits from the advantages of Pareto-, decomposition- and indicator-based approaches. First, Pareto dominance alleviates the selection of dominated solutions. Second, weight vectors assist in the selection of well-diversified and convergent solutions in each generation. Third, if the weight vector fail to differentiate the high-quality parent solutions then the indicator assists the selection process by considering both convergence and diversity. The ability of I_SDE_^+^ to select a set of converged and diverse solutions from unselected ones with respect to a set of already selected solutions is expected to aid the uniform weight vectors in achieving better convergence and diversity.

The rest of this paper is organized as follows. The second section presents the preliminaries. The third section introduces related work and motivation for the current study. The fourth section contains details of HS-MOEA. The fifth section presents experimental setup and comparison results of HS-MOEA with a number of state-of-the-art MOEAs. The last section presents the conclusions and future directions.

## Preliminaries

Generally, MOP is formulated as:

1 $$ \begin{gathered} \min f\left( x \right) = \left( {f_{1} \left( x \right), f_{2} \left( x \right), \ldots ,f_{M} \left( x \right)} \right) \hfill \\ {\text{s.t.}}\,\,x = \left( {x_{1} , x_{2} , \ldots ,x_{D} } \right) \in {\Omega } \subset {\varvec{R}}^{{\varvec{D}}} \hfill \\ \end{gathered} $$

where $x$ represents an $D$ dimensional decision vector in $\Omega$, and $M$ is the number of objectives.

In multi-objective optimization, the following concepts have been well defined and widely applied.

1. Pareto Dominance:
  For any two solutions $x$ and $y$, $x$ is said to dominate $y,$ denoted as $x\prec y$ *iff* ${f}_{i}(x)\le {f}_{i}(y)$ $\forall$ $i\in \{\mathrm{1,2},\dots ,M\}$ and ${f}_{j}(x)<{f}_{j}(y)$ for at least one $j\in \{\mathrm{1,2},\dots ,M\}$.
2. Pareto Optimality:
  A solution ${\mathrm{x}}^{*}$ is said to be Pareto-optimal if there is no other solution $\mathrm{x}\in\Omega$ such that $\mathrm{x}\prec {\mathrm{x}}^{*}$.
3. Pareto-optimal Set (PS):
  It is the set of all Pareto-optimal solutions and is defined as $\mathrm{PS}=\left\{\mathrm{x}\in\Omega \right|\mathrm{x is Pareto optimal}\}$.
4. Pareto-optimal Front (PF):
  It is the set of all Pareto-optimal solutions and is defined as $PF=\left\{f(x)\in {R}^{M}\right|x\in PS\}$*.*
5. Ideal point:
  Ideal point is a vector ${\mathrm{z}}^{*}=({\mathrm{z}}_{1}^{*},{\mathrm{z}}_{2}^{*},\dots ,{\mathrm{z}}_{\mathrm{M}}^{*} )$, which is the infimum of ${f}_{i}$ for each $\mathrm{i}\in \{\mathrm{1,2},\dots ,\mathrm{M}\}$ in the PF.
6. Nadir point:
  Nadir point is a vector ${\mathrm{z}}^{\mathrm{nad}}=({\mathrm{z}}_{1}^{\mathrm{nad}},{\mathrm{z}}_{2}^{\mathrm{nad}},\dots ,{\mathrm{z}}_{\mathrm{M}}^{\mathrm{nad}} )$, which is the supremum of ${f}_{i}$ for each $\mathrm{i}\in \{\mathrm{1,2},\dots ,\mathrm{M}\}$ in the PF.
7. Weight vector:
  A weight vector $w$ is a $M$ dimensional vector $w=({w}_{1},{w}_{2},\dots ,{w}_{M})$ such that ${\sum }_{i=1}^{M}{w}_{i}=1$ and ${w}_{i}\ge 0 \forall i\in \{\mathrm{1,2},\dots ,M\}$. The Normal Boundary Intersection (NBI) method is a systematic approach that places points on a normalized hyper-plane*,* i.e., on a $(M-1)$-dimensional unit simplex. It generates $\left(\genfrac{}{}{0pt}{}{H+M-1}{M-1}\right)$ number of finite weights where $M$ is the number of objective of problem and $H$ is the number of divisions considered along each objective coordinate. Moreover, all the generated points are equally and uniformly distributed in that latex structure.
8. Penalty-based Boundary Intersection (PBI) Operator:
  Let $\mathrm{w}=({\mathrm{w}}_{1},{\mathrm{w}}_{2},\dots ,{\mathrm{w}}_{\mathrm{M}})$ be a direction vector, where $\sum_{\mathrm{i}}{\mathrm{w}}_{\mathrm{i}}=1$. The PBI operator is defined as:
  2 $$ \begin{aligned} g^{pbi} \left( {x,w,z^{*} } \right) &= d_{1} + \theta d_{2} \hfill \\ {\text{where}},\,d_{1} &= \left( {F\left( x \right) - z^{*} } \right)^{T} w/\|w\| \hfill \\ d_{2} &= \| F\left( x \right) - z^{*} - \left( {d_{1} /\|w\|} \right)w\| \hfill \\ \end{aligned} $$
  where $\| \cdot \|$ denotes ${L}_{2}$ norm and $\theta$ is a penalty parameter.

## Related work and motivation

In RV-MOEAs, the objective vector corresponding to each solution is converted into a scalar value based on a series of uniformly distributed weight vectors. To maintain population diversity during the evolution, RV-MOEAs assign the same search space preference to each direction vector. However, the performance of RV-MOEAs strongly depends on the shape of the PF^28^, which is not known in advance. In addition, the size and shape of PF vary over generators. Therefore, it is essential to adapt the weight vectors during the evolution process or employ a secondary selection criterion to aid the uniform weight vectors.

**Figure 1:**
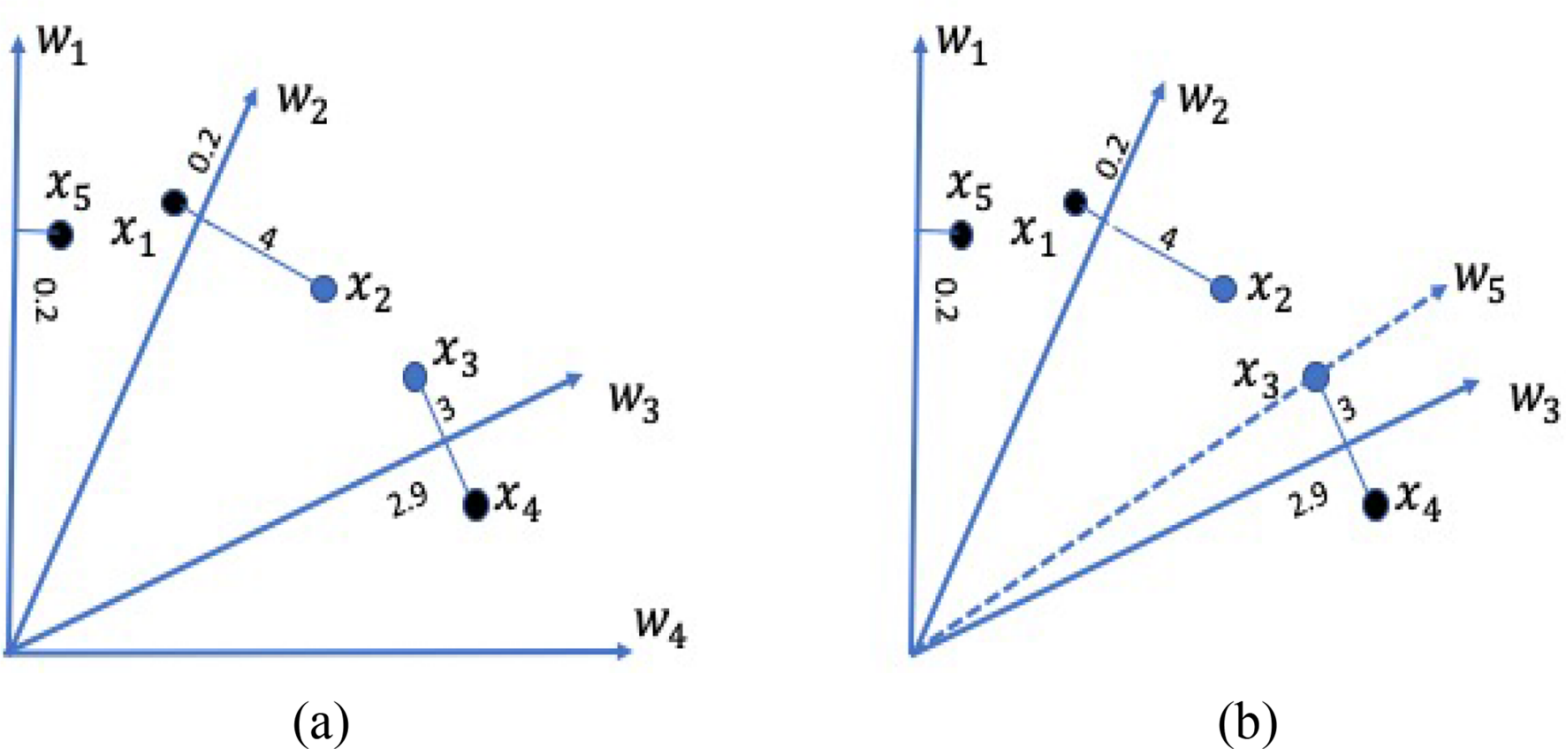
Selection of PMEA.

In the literature, attempts have been made to improve the performance of RV-MOEAs on MaOPs with both the regular and irregular PFs. In NSGA-III^11^, significant changes to the selection operator were performed compared to its predecessor NSGA-II, where the diversity promotion among population members is achieved by a set of well-spread reference points. The employment of reference vectors improves the scalability of the algorithm by reducing the computational complexity that arises due to the increase in the number of objectives. NSGA-III is further modified (referred to as A-NSGA-III^20^) where ineffective reference points are re-allocated based on the distribution and association of the solution. Ineffective reference vectors are the ones that do not have any population members associated with them. RVEA^21^ employs a scalarization approach, termed as Angle Penalized Distance, that assesses convergence by calculating the distance between the candidate solution and the weight vector. In^27^, a weight vector adaptation strategy was employed to enhance the performance of RVEA. TDEA^8^ enhances the convergence of NSGA-III in high dimensional objective spaces by—(1) incorporating a new dominance scheme and (2) employing the aggregation function-based fitness evaluation scheme of MOEA/D. In Polar Metric based Evolutionary Algorithm (PMEA)^23^, a metric inspired from the PBI operator referred to as polar-metric (p-metric) is proposed to measure the convergence and diversity. During environmental selection, a weight vector adjustment strategy is employed to select the well-diversified solutions. The environmental selection of PMEA is demonstrated in Fig. 1 where *x*_*i*_ and *w*_*i*_ represent the solutions and weight vectors, respectively. The values on the perpendicular lines represent the p-metric values of the solutions to the corresponding weight vectors. As in Fig. 1a, PMEA assigns ${x}_{1},{x}_{4}, {x}_{5}$ to ${w}_{2},{w}_{3}, {w}_{1}$, respectively and selects for future evolution. Solutions ${x}_{2}$ and ${x}_{3}$ are not selected. In addition, ${w}_{4}$ does not have any associated solutions and is considered as ineffective. The ineffective weight vector (${w}_{4}$) is re-initialized (${w}_{5}$) to pass through the nearest non-selected solution (*x*_3_) as shown in Fig. 1b. After the re-initialization, solution *x*_3_ associated with weight vector ${w}_{5}$ is selected. In other words, starting with uniform weight vectors, at first, PMEA selects solutions based on p-metric. Later, a weight vector adaptation is made to select the rest of the solutions necessary to create the population for the next generation. However, during the next generation, the weight vectors are uniformly initialized before evaluating the p-metric. From Fig. 1b, it can be noticed that the weight vectors are not well diversified can affect the population diversity during the evolution process. In other words, adapting weights to select the solution in each generation based on the distribution of population is not appropriate.

Motivated by these observations, we propose to combine the advantages of Pareto, decomposition, and indicator methods in a single framework. In the proposed framework, the uniform weight vectors are assisted by I_SDE_^+^ indicator, which serves as a secondary criterion.

## Hybrid selection based multi/many-objective evolutionary algorithm (HS-MOEA)

In this section, Hybrid Selection based Multi/Many-Objective Evolutionary Algorithm (HS-MOEA) is introduced. The different steps of HS-MOEA are detailed below.

### Initialization

A set of uniform weight vectors ($w$) are generated using the NBI method, then subsequently, a population of size $N$ ($\left|w\right|$) is initialized within the permissible boundaries as shown in Line 01 of Algorithm 1.

### Mating selection and offspring generation

I_SDE_^+^ indicator values corresponding to each solution in the population (P_*t*_), corresponding to the *t*th iteration is evaluated using Eq. (3) (Line 04, Algorithm 1). The solutions with the highest I_SDE_^+^ values are considered to be better. Using I_SDE_^+^ values, binary tournament selection is performed to generate the mating pool (Line 05, Algorithm 1). Then, the offspring population is generated using the variation operators such as polynomial mutation^6^ and simulated binary crossover^6^ (Line 06, Algorithm 1).

In HS-MOEA, the mating selection is performed using I_SDE_^+^ indicator. The indicator values corresponding to each solution in the population is evaluated using Eq. (3) (Line 04, Algorithm 1).

3 $$ {\text{I}}_{{{\text{SDE}}}}^{ + } \left( { x} \right) = \begin{array}{*{20}c} {min} \\ {y \in P_{SB\left( x \right)} , x \ne y} \\ \end{array} \left\{ {dist\left( {F\left( x \right),F\left( {y_{1}^{^{\prime}} } \right)} \right), dist\left( {F\left( x \right),F\left( {y_{2}^{^{\prime}} } \right)} \right), \ldots ,dist\left( {F\left( x \right),F\left( {y_{{N_{SB} \left( x \right)}}^{^{\prime}} } \right)} \right)} \right\} $$

where $P_{SB\left( x \right)} \in P_{t}$ and $y \in P_{SB} \left( x \right)$, such that $SB\left(y\right)<SB\left(x\right).$ $SB$ represent the sum of normalized objectives. ${N}_{SB(x)}$ represents the size of ${P}_{SB(x)}$.

4 $$ F\left( {y_{i}^{^{\prime}} \left( j \right)} \right) = \left\{ {\begin{array}{*{20}c} {F\left( {x\left( j \right)} \right)} & { if F\left( {y_{i} \left( j \right)} \right) < F\left( {x\left( j \right)} \right)} \\ {F\left( {y_{i} \left( j \right)} \right)} & { otherwise} \\ \end{array} } \right.\quad {\text{for}}\,\,j = 1,2, \ldots ,M $$

The solutions with the highest I_SDE_^+^ values are considered to be better. Then, the binary tournament selection is performed based on I_SDE_^+^ values to generate the matting pool (Line 05, Algorithm 1). After the mating selection, the offspring population is generated using the variation operators such as mutation and crossover, as shown in Line 06, Algorithm 1. In the current, the mutation and crossover operators employed are Polynomial Mutation (PM)^2^ and Simulated Binary Crossover (SBX)^2^.

### Normalization

Normalization (Line 07, Algorithm 1) is an essential tool to map the unscaled search space to scaled one so as to characterize the badly scaled objectives. In HS-MOEA, the normalization (of the *j*^th^ population member is given in Eq. (5).

5 $$ F_{i}^{j} = \frac{{f_{i}^{j} - z_{i}^{*} }}{{z_{i}^{nad} - z_{i}^{*} }}, \forall i = 1,2, \ldots ,M $$

where, ${z}_{i}^{*}$ and ${z}_{i}^{nad}$ are considered as the lowest and highest values of ${i}^{th}$ objective function.
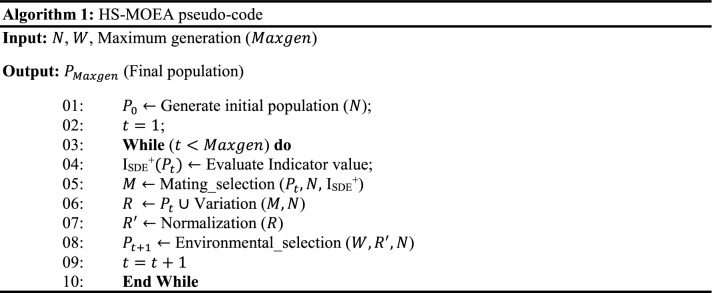



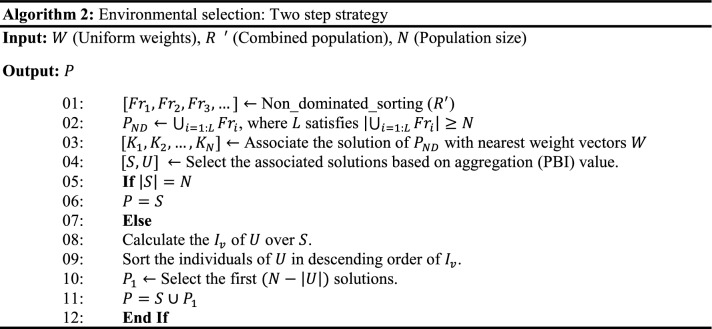



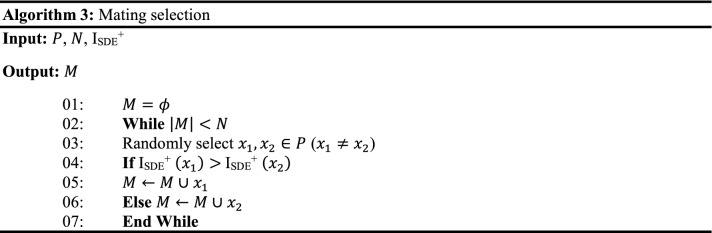


### Environmental Selection

The environmental selection selects a set of *N* converged but diversified solutions from a combined population (*R*) of size $2N$ (Line 08, Algorithm 1). The working mechanism is detailed in Algorithm 2. Non-dominated sorting^6^ (Lines 01 ~ 02, Algorithm 2) is performed to classify the population $R$ into several fronts (*Fr*) and identify the population ${P}^{ND}=\bigcup_{l=1:L}{Fr}_{l}$ (where $L$ satisfies $\left|\bigcup_{l=1:L}{Fr}_{l}\right|\ge N$ and $\left|\bigcup_{l=1:L-1}{Fr}_{l}\right|<N)$.

#### Association

In HS-MOEA, the association procedure (Line 04, Algorithm 2) is performed in the normalized objective space, where the ideal point $${z}^{*}$$ is shifted to origin. At each generation, the individuals of the $${P}_{t}^{ND}$$ population is associated with the reference vectors ($$w)$$. For the association operator, the norm of each solution *x* in $${P}_{t}^{ND}$$ is evaluated as:

6 $$ norm\left( {F\left( x \right)} \right) = \sqrt {\mathop \sum \limits_{i = 1}^{M} F_{i} \left( x \right)^{2} } $$

Then, the angle between $F(x)$ and ${w}^{i}$ is defined as:

7 $$ angle\left( {F\left( x \right),w^{i} } \right) = {\text{arccos}}\left| {\frac{{F\left( x \right) \circ w^{i} }}{{norm\left( {F\left( x \right)} \right) \cdot norm\left( {w^{i} } \right)}}} \right| $$

where, $F\left(x\right)\circ {w}^{i}=\sum_{i=1}^{M}{F}_{i}(x)\cdot {w}_{i}^{j}$ is the dot product of $F\left(x\right)=\left({F}_{1}\left(x\right), {F}_{2}\left(x\right),\dots , {F}_{M}\left(x\right)\right),$ and ${w}^{i}=({w}_{1}^{i}, {w}_{2}^{i},\dots , {w}_{M}^{i})$.

**Figure 2:**
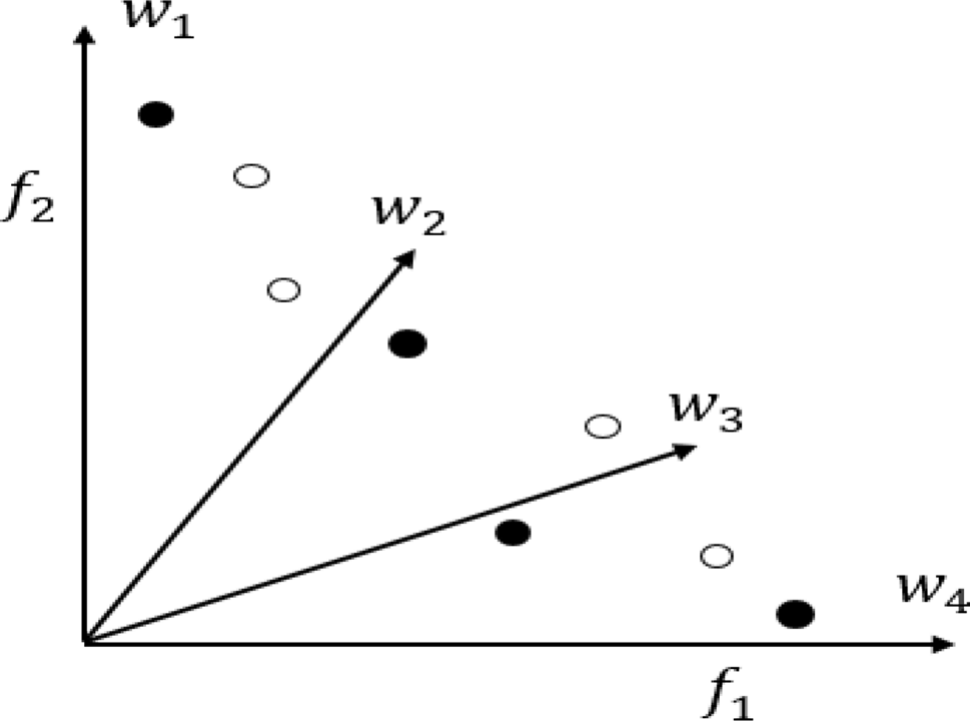
Illustration of the association operator using an acute angle.

During association, each solution is assigned to its closest reference vector. *K*_*i*_ is the number of solutions associated with the weight vector *w*_*i*_ during the association process, which can range from $0$ to $N$. Figure 2 represents the association operator, where the filled circles are the associated solutions with the corresponding nearest weight vector.

#### Two step selection

For each $x$ and $y$ $\in$ ${K}_{i}$, $x$ is preferred to $y$ iff $PBI\left(x,{w}^{i}\right)<PBI(y,{w}^{i})$ where $PBI\left(x,w\right)={d}_{1}+\theta {d}_{2}$, ${d}_{2}=$ perpendicular distance of $x$ over $w$, ${d}_{1}=$ distance between the origin and the projected point of $x$ over $w$, $\theta =$ penalty parameter. PBI refers to the penalty-based boundary intersection^16^.

First, select one solution from each non-empty ${K}_{i}$ $\forall i=\mathrm{1,2},\dots ,N$ based on $PBI$ value and save them in $S$ (referred to as a set of selected solutions). The remaining solutions are stored in $U$ (referred to as a set of unselected solutions). If the size of $S$ is $N$ then the whole set $S$ is declared as a parent population of the next generation (Line 06, Algorithm 2); otherwise, go for the second round of selection. In the second round, $(N-\left|S\right|)$ solutions are to be selected from $U$ using I_SDE_^+^ indicator. For each $x\in U$ the values of I_SDE_^+^ referred to as I^U^_SDE_^+^ is calculated (Line 08, Algorithm 2). To evaluate the indicator, the solutions in *U* are sorted in the ascending order of the normalized sum of objectives (SB) (Line 09, Algorithm 2). The solution with the least SB is assigned the highest possible indicator value of one. To evaluate the I^U^_SDE_^+^ of a given solution $x\in U$, the solutions in *U* that are better in convergence with the least SB compared to *x* and solutions in set S are shifted as in Eq. (8). Then $(N-\left|S\right|)$ solutions from *U* with the largest I^U^_SDE_^+^ are selected (*P*_1_) (Line 10, Algorithm 2) and added to S (Line 11, Algorithm 2), which becomes the population (*P*) for the next generation.

8 $$ I_{SDE}^{U + } = \begin{array}{*{20}c} {\min } \\ {y \in L, x \ne y} \\ \end{array} \left\{ {dist\left( {F\left( x \right),F\left( {y_{1}^{^{\prime}} } \right)} \right), dist\left( {F\left( x \right),F\left( {y_{2}^{^{\prime}} } \right)} \right), \ldots ,dist\left( {F\left( x \right),F\left( {y_{\left| A \right|}^{^{\prime}} } \right)} \right)} \right\} $$

where $A$ is (${U}_{SB}\left(x\right)+S)$. ${U}_{SB}\left(x\right)\in U$ and $y\in A.$ For all $y\in {U}_{SB}\left(x\right)$ such that $SB\left(y\right)<SB\left(x\right)$, $S$ and *U* represent a set of selected and unselected solutions respectively by weight vector association.

In other words, the use of I_SDE_^+^ indicator enables the selection of converged, yet diverse solutions with respect to the already selected solutions (*S*). First, Pareto dominance alleviates the selection of dominated solutions. Second, weight vectors assist in the selection of well-diversified and convergent solutions in each generation. Third, if the weight vector fails to differentiate the high-quality parent solutions, then the indicator assists the selection process by considering both convergence and diversity. The advantage of employing I_SDE_^+^ is that it enables the selection of the solutions considering the solutions that are already selected through weight vector association in the second step. In other words, HS-MOEA gets benefitted from both the reference vectors and indicators.

### Computational complexity

The non-dominated sorting (Line 01, Algorithm 2) of a population of size 2* N* having *M*-dimensional objective vectors requires $O(Nlo{g}^{M-2}N )$ computations^11^. All operations in Algorithm 2 in associating a maximum of $2N$ population members to $(H\approx N)$ reference points would require $O(MNH)$. Thereafter, for calculating the I_SDE_^+^ of maximum $N$ population members would require $O({N}^{2})$. Therefore, the computational complexity of one generation of HS-MOEA is $O({N}^{2}lo{g}^{M-2}N )$ or $O(M{N}^{2})$, whichever is larger.

## Experimental setup, results and discussion

Experiments were conducted on 16 scalable test problems from DTLZ^29^ and WFG^30^, test suites comprising of 7 and 9 problems, respectively. For each test problem, 2-, 4-, 6-, 8- and 10-objectives are considered. The parameter values employed are present in Table 1. On each instance, 30 independent runs were performed for each algorithm on a PC with a 3.30 GHz Intel (R) Core (TM) i7- 8700 CPU and Windows 10 Pro 64-bit operating system with 16 GB RAM. As a stopping criterion, the maximum number of generations for DTLZ1 and WFG2 is set to 700 and for DTLZ3 and WFG1 it is set as 1000. For the other problems (DTL2, DTLZ4–7, and WFG3–9) it is set to 250. All algorithms considered employ population size (*N*) of 100, 165, 182, 240 and 275 for 2-, 4-, 6-, 8-, 10-objectives, respectively^13^. Simulated binary crossover and polynomial mutation with distribution indices and probabilities set to ${n}_{m}=20$, ${n}_{c}=20$, ${p}_{c}=1.0$ and ${p}_{m}=1/D$, respectively, are employed.

**Table 1:** Parameter settings for DTLZ and WFG test suites.

Parameter	DTLZ1	DTLZ2-DTLZ6	DTLZ7	WFG1–WFG9
k	5	10	20	–
M	2, 4, 6, 8, 10			2, 4, 6, 8, 10
K	–			4, 6, 10, 7, 9
L	–			10
D	M − 1 + K			K + L

*K* DTLZ problem specific parameter, *M* number of objectives, *K* position vector, *L* distance vector, *D* number of variables.

In order to compare the efficiency of HS-MOEA with the state-of-the-art algorithms such as PMEA, TDEA, RVEA, NSGAIII, MOEA/D, I_SDE_^+^, IBEA, ONEBYONE^31^, a quantitative indicator, namely HyperVolume (HV) is being employed. The larger value of HV implies the superiority of the algorithm. The experimental results (mean and standard deviation values of normalized HV) on benchmark suites are presented in Table 2. In addition, the statistical tests (t-test) at a 5% significance level were conducted to compare the significance of the difference between the mean metric values yielded by HS-MOEA and state-of-the-art algorithms. The signs “ + ”, “−” and “≈” against the HV values indicate that the HS-MOEA is statistically “better”, “worse” and “comparable” with the corresponding algorithm, respectively. The last row of Table 2 represents the overall performance of HS-MOEA in terms of the number of instances it is better (Win-W), comparable (Tie-T) and worst (Loss-L) with respect to the corresponding algorithm.

**Table 2:** Comparison of HV and statistical results on DTLZ and WFG test problems (“+”—win, “≈”—TIE, “−”—loss).

#	M	HS-MOEA	PMEA	TDEA	RVEA	NSGAIII	MOEA/D	MOEA/DD	I_SDE_^+^	1BY1EA
DTLZ1	2	4.94E−1 (6.06E−4)	4.94E−1 (6.44E−4)−	4.95E−1 (4.84E−4)−	4.95E−1 (7.42E−4)−	4.95E−1 (6.16E−4)−	4.95E−1 (6.88E−4)−	4.95E−1 (5.36E−4)−	4.94E−1 (6.42E−4)≈	4.82E−1 (6.94E−3) +
	4	9.13E−1 (1.51E−3)	5.73E−1 (1.62E−1) +	9.19E−1 (3.53E−4)−	9.19E−1 (3.97E−4)−	9.19E−1 (4.03E−4)−	9.19E−1 (5.52E−4)−	9.19E−1 (4.41E−4)−	9.10E−1 (1.68E−3) +	8.66E−1 (1.02E−2) +
	6	9.68E−1 (4.29E−3)	3.22E−1 (8.93E−2) +	9.82E−1 (2.97E−3)−	9.83E−1 (1.98E−4)−	9.83E−1 (2.79E−4)−	9.82E−1 (4.18E−4)−	9.82E−1 (2.28E−4)−	9.75E−1 (2.40E−3)−	9.39E−1 (7.84E−3) +
	8	9.88E−1 (2.16E−3)	3.52E−1 (7.72E−2) +	9.64E−1 (1.48E−1)≈	9.95E−1 (1.98E−4)−	9.94E−1 (2.45E−3)−	9.83E−1 (4.02E−3) +	9.86E−1 (2.30E−3) +	9.92E−1 (9.42E−4)−	9.72E−1 (3.02E−3) +
	10	9.92E−1 (2.09E−3)	6.10E−1 (8.00E−2) +	9.68E−1 (1.48E−1)≈	9.99E−1 (4.01E−5)−	9.88E−1 (4.14E−2)≈	9.97E−1 (6.56E−4)−	9.94E−1 (2.47E−3)−	9.96E−1 (7.55E−4)−	9.86E−1 (2.35E−3) +
DTLZ2	2	2.09E−1 (4.80E−4)	2.10E−1 (4.06E−4)−	2.10E−1 (4.36E−4)−	2.09E−1 (8.60E−4) +	2.10E−1 (4.20E−4)−	2.10E−1 (3.70E−4)−	2.10E−1 (4.54E−4)−	2.10E−1 (3.78E−4)−	2.10E−1 (3.20E−4)−
	4	5.84E−1 (5.39E−4)	5.84E−1 (5.72E−4)≈	5.83E−1 (4.78E−4) +	5.82E−1 (5.69E−4) +	5.82E−1 (5.57E−4) +	5.83E−1 (5.71E−4) +	5.83E−1 (4.55E−4) +	5.79E−1 (2.69E−3) +	5.71E−1 (2.85E−3) +
	6	7.60E−1 (5.61E−4)	7.60E−1 (5.58E−4)≈	7.57E−1 (7.95E−4) +	7.57E−1 (6.38E−4) +	7.53E−1 (1.26E−3) +	7.56E−1 (6.98E−4) +	7.60E−1 (4.75E−4)≈	7.68E−1 (3.43E−3)−	7.39E−1 (6.51E−3) +
	8	8.66E−1 (4.72E−4)	8.63E−1 (7.90E−4) +	8.58E−1 (1.03E−3) +	8.59E−1 (8.82E−4) +	8.42E−1 (3.17E−2) +	8.49E−1 (2.92E−3) +	8.51E−1 (3.23E−3) +	8.84E−1 (3.17E−3)−	8.56E−1 (4.12E−3) +
	10	9.48E−1 (2.41E−4)	9.48E−1 (3.20E−4) +	9.45E−1 (4.40E−4) +	9.46E−1 (3.14E−4) +	9.08E−1 (4.69E−2) +	9.48E−1 (5.51E−4) +	9.45E−1 (5.93E−4) +	9.48E−1 (1.56E−3) +	9.21E−1 (3.32E−3) +
DTLZ3	2	2.09E−1 (9.35E−4)	2.09E−1 (1.02E−3)≈	2.09E−1 (1.06E−3)≈	2.09E−1 (1.50E−3)≈	2.09E−1 (1.05E−3) +	2.08E−1 (2.07E−3) +	1.76E−1 (5.64E−2) +	2.09E−1 (1.14E−3) +	1.24E−1 (7.82E−2) +
	4	5.96E−1 (2.81E−3)	3.71E−1 (1.03E−1) +	5.91E−1 (3.90E−3) +	5.93E−1 (2.89E−3) +	5.92E−1 (2.80E−3) +	5.76E−1 (1.52E−2) +	5.87E−1 (5.72E−3) +	5.90E−1 (3.23E−3) +	5.83E−1 (4.07E−3) +
	6	8.19E−1 (2.18E−3)	1.51E−1 (1.70E−1) +	8.04E−1 (6.94E−3) +	8.10E−1 (2.75E−3) +	7.98E−1 (3.42E−2) +	7.87E−1 (6.10E−2) +	1.09E−1 (1.81E−1) +	8.12E−1 (5.78E−3) +	6.37E−1 (3.01E−1) +
	8	9.75E−1 (5.79E−4)	1.93E−1 (1.33E−1) +	9.42E−1 (6.24E−2) +	9.69E−1 (1.30E−3) +	9.21E−1 (1.38E−1) +	9.34E−1 (9.72E−2) +	6.30E−1 (3.22E−1) +	9.74E−1 (1.10E−3) +	9.71E−1 (9.46E−4) +
	10	1.00E + 0 (0.00E + 0)	4.83E−1 (1.98E−1) +	1.00E + 0 (0.00E + 0)≈	1.00E + 0 (0.00E + 0)≈	1.00E + 0 (3.65E−7)≈	9.99E−1 (1.96E−3) +	1.00E + 0 (2.49E−6) +	1.00E + 0 (0.00E + 0)≈	1.00E + 0 (0.00E + 0)≈
DTLZ4	2	1.54E−1 (9.42E−2)	1.81E−1 (7.23E−2)≈	1.89E−1 (6.41E−2)−	2.09E−1 (1.02E−3)−	1.82E−1 (7.27E−2)≈	1.74E−1 (7.93E−2)≈	2.10E−1 (4.42E−4)−	2.10E−1 (4.43E−4)−	1.96E−1 (5.33E−2)−
	4	5.91E−1 (5.43E−4)	5.91E−1 (6.26E−4) +	5.90E−1 (6.59E−4) +	5.90E−1 (4.94E−4) +	5.77E−1 (4.63E−2)≈	3.89E−1 (1.41E−1) +	5.90E−1 (6.01E−4) +	5.85E−1 (2.27E−3) +	5.83E−1 (8.38E−3) +
	6	7.92E−1 (4.84E−4)	7.92E−1 (5.05E−4) +	7.90E−1 (7.30E−4) +	7.88E−1 (1.27E−2) +	7.78E−1 (2.37E−2) +	6.26E−1 (1.12E−1) +	7.86E−1 (1.63E−2) +	7.93E−1 (3.44E−3)−	7.86E−1 (1.61E−2) +
	8	9.03E−1 (3.12E−4)	9.01E−1 (6.72E−4) +	8.98E−1 (7.75E−4) +	8.99E−1 (6.00E−4) +	8.86E−1 (2.50E−2) +	7.94E−1 (8.24E−2) +	8.97E−1 (1.08E−3) +	9.11E−1 (2.27E−3)−	9.09E−1 (1.55E−3)−
	10	9.64E−1 (1.87E−4)	9.65E−1 (2.57E−4)−	9.64E−1 (2.87E−4)≈	9.64E−1 (3.28E−4)≈	9.58E−1 (1.54E−2) +	8.44E−1 (8.11E−2) +	9.64E−1 (2.68E−4) +	9.60E−1 (2.07E−3) +	9.58E−1 (1.13E−3) +
DTLZ5	2	2.10E−1 (5.00E−4)	2.10E−1 (2.93E−4)−	2.10E−1 (3.26E−4)−	2.09E−1 (1.00E−3) +	2.10E−1 (3.12E−4)−	2.10E−1 (4.03E−4)−	2.10E−1 (4.16E−4)−	2.10E−1 (5.23E−4)−	2.10E−1 (3.48E−4)−
	4	7.25E−1 (1.56E−3)	7.21E−1 (1.73E−3) +	7.14E−1 (4.07E−3) +	6.86E−1(3.52E−3) +	7.12E−1 (6.56E−3) +	7.24E−1 (5.06E−4) +	7.15E−1 (1.49E−3) +	7.25E−1 (1.16E−3)≈	7.19E−1 (1.56E−3) +
	6	8.37E−1 (3.07E−3)	8.24E−1 (1.02E−2) +	8.16E−1 (7.34E−3) +	7.75E−1 (6.82E−3) +	8.06E−1 (2.50E−2) +	7.83E−1 (8.08E−3) +	7.90E−1 (1.02E−2) +	8.33E−1 (3.82E−3) +	7.75E−1 (4.01E−3) +
	8	8.47E−1 (2.31E−3)	8.20E−1 (1.06E−2) +	8.21E−1 (8.57E−3) +	7.10E−1 (1.92E−2) +	8.08E−1 (1.63E−2) +	7.72E−1 (2.58E−3) +	7.93E−1 (7.60E−3) +	8.44E−1 (3.04E−3) +	7.80E−1 (4.41E−3) +
	10	8.61E−1 (2.16E−3)	7.85E−1 (1.61E−2) +	8.48E−1 (4.49E−3) +	7.98E−1 (1.10E−2) +	8.21E−1 (2.26E−2) +	7.94E−1 (1.28E−3) +	8.21E−1 (4.04E−3) +	8.57E−1 (3.96E−3) +	8.03E−1 (4.54E−3) +
DTLZ6	2	2.10E−1 (4.48E−4)	2.10E−1 (3.19E−4) +	2.10E−1 (4.44E−4)≈	2.10E−1 (3.38E−4)≈	2.10E−1 (4.44E−4)≈	2.10E−1 (3.87E−4)≈	2.10E−1 (4.33E−4)≈	2.10E−1 (5.44E−4)≈	2.10E−1 (3.51E−4)−
	4	9.13E−1 (4.90E−4)	9.07E−1 (1.13E−2) +	9.08E−1 (1.52E−3) +	9.01E−1 (2.99E−3) +	9.06E−1 (3.18E−3) +	9.11E−1 (1.39E−3) +	9.09E−1 (2.22E−3) +	9.13E−1 (6.10E−4) +	9.14E−1 (3.43E−4)−
	6	9.80E−1 (9.67E−4)	9.60E−1 (2.62E−2) +	9.77E−1 (1.83E−3) +	9.54E−1 (1.89E−2) +	9.71E−1 (8.01E−3) +	9.42E−1 (1.82E−2) +	9.63E−1 (3.45E−3) +	9.79E−1 (1.16E−3) +	9.72E−1 (1.88E−3) +
	8	9.84E−1 (2.66E−3)	9.60E−1 (2.34E−2) +	9.83E−1 (9.08E−4) +	9.65E−1 (2.22E−2) +	9.62E−1 (1.97E−2) +	9.56E−1 (1.46E−3) +	9.75E−1 (7.20E−3) +	9.84E−1 (6.39E−4)≈	9.64E−1 (2.05E−2) +
	10	9.80E−1 (6.50E−3)	9.32E−1 (2.64E−2) +	9.82E−1 (7.92E−4)≈	9.66E−1 (1.19E−2) +	9.58E−1 (2.40E−2) +	9.54E−1 (8.94E−4) +	9.75E−1 (3.96E−3) +	9.82E−1 (1.24E−3)−	9.46E−1 (2.21E−2) +
DTLZ7	2	1.41E−1 (4.94E−4)	1.41E−1 (3.54E−4)≈	1.41E−1 (4.02E−4)≈	1.26E−1 (3.28E−3) +	1.41E−1 (3.26E−4)≈	1.19E−1 (2.26E−2) +	1.37E−1 (5.18E−4) +	1.41E−1 (6.65E−4) +	6.56E−2 (5.31E−3) +
	4	1.98E−1 (4.52E−3)	1.94E−1 (3.55E−3) +	1.81E−1 (6.63E−3) +	1.59E−1 (6.20E−3) +	1.67E−1 (4.25E−3) +	9.08E−2 (1.15E−2) +	4.51E−2 (2.50E−2) +	1.89E−1 (6.40E−3) +	1.65E−1 (7.86E−3) +
	6	1.86E−1 (3.45E−3)	1.82E−1 (1.79E−3) +	1.12E−1 (2.22E−2) +	1.23E−1 (9.59E−3) +	1.36E−1 (5.54E−3) +	1.94E−3 (1.11E−3) +	5.20E−2 (1.22E−2) +	1.76E−1 (5.50E−3) +	6.72E−2 (1.22E−2) +
	8	1.60E−1 (3.48E−3)	1.30E−1 (4.84E−3) +	1.08E−1 (2.10E−2) +	9.12E−2 (1.14E−2) +	1.10E−1 (9.86E−3) +	1.51E−4 (7.10E−5) +	2.08E−2 (1.93E−2) +	1.54E−1 (7.92E−3) +	2.67E−2 (9.98E−3) +
	10	1.29E−1 (3.24E−3)	1.07E−1 (3.26E−3) +	1.17E−1 (6.22E−3) +	6.04E−2 (1.16E−2) +	1.04E−1 (6.11E−3) +	7.74E−4 (1.25E−3) +	2.73E−4 (1.60E−4) +	1.20E−1 (1.67E−2) +	2.01E−2 (8.16E−3) +
WFG1	2	6.08E−1 (1.65E−2)	6.20E−1 (6.26E−3)−	6.28E−1 (1.90E−3)−	6.26E−1 (7.82E−3)−	6.26E−1 (2.95E−3)−	5.59E−1 (1.42E−3) +	5.86E−1 (1.20E−2) +	6.07E−1 (1.45E−2)≈	6.19E−1 (7.42E−3)−
	4	9.47E−1 (1.01E−2)	9.40E−1 (6.28E−3) +	9.85E−1 (6.59E−4)−	9.85E−1 (5.46E−3)−	9.87E−1 (3.37E−4)−	8.42E−1 (1.24E−2) +	9.24E−1 (3.37E−2) +	9.60E−1 (6.22E−3)−	9.64E−1 (4.50E−3)−
	6	9.72E−1 (7.92E−3)	9.65E−1 (8.28E−3) +	9.90E−1 (1.54E−3)−	9.93E−1 (5.18E−3)−	9.99E−1 (2.71E−4)−	8.50E−1 (1.74E−2) +	9.12E−1 (4.35E−2) +	9.77E−1 (6.80E−3)−	9.89E−1 (6.14E−3)−
	8	9.86E−1 (4.47E−3)	9.42E−1 (4.98E−2) +	9.87E−1 (1.55E−2)≈	9.86E−1 (2.78E−2)≈	9.94E−1 (5.48E−3)−	7.92E−1 (2.54E−2) +	9.24E−1 (3.76E−2) +	9.91E−1 (2.45E−3)−	9.89E−1 (3.70E−3)−
	10	9.90E−1 (2.30E−3)	9.74E−1 (3.11E−2) +	9.93E−1 (1.61E−3)−	9.91E−1 (6.40E−3)≈	9.98E−1 (1.75E−3)−	6.36E−1 (1.79E−1) +	8.62E−1 (6.35E−2) +	9.91E−1 (1.84E−3)≈	9.88E−1 (4.74E−3) +
WFG2	2	5.41E−1 (1.70E−3)	5.41E−1 (1.52E−3)≈	5.51E−1 (1.28E−3)−	5.28E−1 (9.37E−3) +	5.44E−1 (4.53E−3)−	3.50E−1 (6.58E−2) +	5.23E−1 (2.31E−2) +	5.40E−1 (2.72E−3) +	5.40E−1 (1.32E−2)≈
	4	9.43E−1 (4.16E−3)	9.42E−1 (5.20E−3)≈	9.82E−1 (1.04E−3)−	9.63E−1 (3.82E−3)−	9.78E−1 (2.78E−3)−	8.31E−1 (2.22E−2) +	8.99E−1 (1.44E−2) +	9.37E−1 (1.10E−2) +	9.44E−1 (7.76E−3)≈
	6	9.41E−1 (9.38E−3)	9.42E−1 (1.07E−2)≈	9.77E−1 (1.46E−2)−	9.60E−1 (1.21E−2)−	9.92E−1 (2.83E−3)−	8.13E−1 (5.59E−2) +	9.05E−1 (3.17E−2) +	9.36E−1 (1.59E−2)≈	9.75E−1 (5.37E−3)−
	8	9.54E−1 (7.21E−3)	9.57E−1 (7.93E−3)≈	9.85E−1 (1.75E−2)−	9.33E−1 (2.01E−2) +	9.94E−1 (3.18E−3)−	7.42E−1 (3.76E−2) +	8.13E−1 (4.73E−2) +	9.46E−1 (1.32E−2) +	9.81E−1 (8.60E−3)−
	10	9.51E−1 (2.05E−2)	9.49E−1 (1.08E−2)≈	9.79E−1 (9.43E−3)−	9.52E−1 (1.22E−2)≈	9.96E−1 (3.11E−3)−	7.24E−1 (6.52E−2) +	9.07E−1 (2.08E−2) +	9.47E−1 (1.47E−2)≈	9.81E−1 (7.41E−3)−
WFG3	2	4.95E−1 (5.86E−4)	4.94E−1 (9.35E−4)≈	4.95E−1 (8.27E−4)−	4.92E−1 (1.62E−3) +	4.95E−1 (5.87E−4)−	4.94E−1 (1.01E−3)≈	4.96E−1 (1.77E−3)−	4.94E−1 (5.29E−4) +	4.83E−1 (5.88E−3) +
	4	4.91E−1 (1.63E−2)	5.60E−1 (3.45E−2)−	4.55E−1 (1.08E−2) +	4.80E−1 (2.72E−2) +	4.82E−1 (2.89E−2)≈	6.04E−1 (4.57E−2)−	5.31E−1 (3.87E−2)−	4.64E−1 (1.05E−2) +	4.24E−1 (4.48E−2) +
	6	4.99E−1 (1.53E−2)	6.35E−1 (2.88E−2)−	4.39E−1 (2.18E−2) +	5.22E−1 (7.26E−2)−	4.87E−1 (1.70E−2) +	6.52E−1 (3.04E−2)−	5.36E−1 (1.49E−2)−	4.57E−1 (2.63E−2) +	4.53E−1 (6.65E−2) +
	8	5.52E−1 (3.74E−2)	5.42E−1 (6.34E−2)≈	2.49E−1 (5.88E−2) +	5.15E−1 (1.14E−1)≈	4.71E−1 (4.39E−2) +	6.77E−1 (5.24E−2)−	4.29E−1 (2.29E−2) +	4.35E−1 (5.10E−2) +	4.94E−1 (9.65E−2) +
	10	6.24E−1 (4.22E−2)	6.16E−1 (6.79E−2)≈	2.95E−1 (6.51E−2) +	8.17E−1 (1.22E−1)−	5.62E−1 (3.54E−2) +	7.72E−1 (1.78E−1)−	4.37E−1 (2.23E−2) +	4.25E−1 (5.90E−2) +	5.80E−1 (1.81E−1)≈
WFG4	2	2.13E−1 (2.70E−3)	2.14E−1 (1.92E−3)≈	2.14E−1 (1.93E−3)≈	1.94E−1 (4.51E−3) +	2.14E−1 (1.89E−3)≈	2.16E−1 (4.98E−3)−	2.17E−1 (3.30E−3)−	2.15E−1 (3.81E−3)−	2.14E−1 (2.79E−3)−
	4	5.75E−1 (1.99E−3)	5.71E−1 (2.23E−3) +	5.66E−1 (2.44E−3) +	5.64E−1 (2.79E−3) +	5.63E−1 (3.53E−3) +	5.36E−1 (5.34E−3) +	5.41E−1 (3.33E−3) +	5.74E−1 (2.92E−3)≈	4.30E−1 (1.83E−2) +
	6	7.31E−1 (3.46E−3)	7.21E−1 (3.19E−3) +	7.17E−1 (4.93E−3) +	6.95E−1 (1.35E−2) +	6.93E−1 (4.47E−2) +	3.37E−1 (1.70E−2) +	6.37E−1 (1.36E−2) +	7.47E−1 (3.75E−3)−	4.54E−1 (2.36E−2) +
	8	8.26E−1 (2.24E−3)	8.02E−1 (6.64E−3) +	8.04E−1 (4.70E−3) +	7.79E−1 (2.02E−2) +	7.99E−1 (1.17E−2) +	1.96E−1 (2.59E−2) +	7.03E−1 (1.88E−2) +	8.35E−1 (4.87E−3)−	5.40E−1 (2.92E−2) +
	10	9.01E−1 (3.04E−3)	8.89E−1 (5.97E−3) +	8.87E−1 (4.37E−3) +	8.45E−1 (1.72E−2) +	8.81E−1 (1.23E−2) +	1.98E−1 (3.65E−2) +	6.38E−1 (2.82E−2) +	8.75E−1 (6.31E−3) +	5.37E−1 (1.70E−2) +
WFG5	2	2.26E−1 (8.95E−4)	2.26E−1 (1.41E−3)−	2.27E−1 (1.39E−3)−	2.17E−1 (3.42E−3) +	2.28E−1 (1.31E−3)−	2.27E−1 (2.07E−3)−	2.31E−1 (2.48E−3)−	2.26E−1 (1.08E−3)≈	2.24E−1 (1.93E−3) +
	4	5.73E−1 (3.76E−3)	5.69E−1 (3.29E−3) +	5.62E−1 (4.94E−3) +	5.66E−1 (3.17E−3) +	5.65E−1 (2.92E−3) +	5.34E−1 (1.69E−2) +	5.44E−1 (3.98E−3) +	5.70E−1 (4.98E−3) +	4.56E−1 (1.74E−2) +
	6	7.23E−1 (1.95E−3)	7.14E−1 (2.36E−3) +	7.13E−1 (3.24E−3) +	7.06E−1 (8.57E−3) +	7.11E−1 (3.62E−3) +	3.52E−1 (2.27E−2) +	6.56E−1 (1.12E−2) +	7.39E−1 (3.04E−3)−	4.84E−1 (2.42E−2) +
	8	8.34E−1 (2.06E−3)	8.07E−1 (3.36E−3) +	8.04E−1 (3.79E−3) +	7.92E−1 (7.28E−3) +	8.03E−1 (4.19E−3) +	2.91E−1 (2.43E−2) +	6.81E−1 (2.47E−2) +	8.49E−1 (4.18E−3)−	5.43E−1 (3.10E−2) +
	10	9.08E−1 (1.69E−3)	8.95E−1 (2.38E−3) +	8.92E−1 (2.53E−3) +	8.67E−1 (1.16E−2) +	8.90E−1 (2.28E−3) +	2.58E−1 (2.22E−2) +	5.90E−1 (3.07E−2) +	8.94E−1 (5.53E−3) +	5.43E−1 (2.85E−2) +
WFG6	2	2.15E−1 (8.57E−3)	2.13E−1 (6.18E−3)≈	2.13E−1 (3.00E−3)≈	1.91E−1 (6.71E−3) +	2.18E−1 (1.01E−2)≈	2.53E−1 (1.98E−2)−	2.24E−1 (1.05E−2)−	2.17E−1 (6.65E−3)≈	2.11E−1 (7.89E−3) +
	4	5.76E−1 (3.00E−3)	5.71E−1 (3.61E−3) +	5.68E−1 (3.93E−3) +	5.64E−1 (4.38E−3) +	5.65E−1 (7.01E−3) +	4.87E−1 (4.27E−2) +	5.42E−1 (5.73E−3) +	5.78E−1 (2.52E−3)−	4.40E−1 (1.78E−2) +
	6	7.39E−1 (8.88E−3)	7.29E−1 (7.39E−3) +	7.25E−1 (1.37E−2) +	7.22E−1 (1.40E−2) +	7.19E−1 (1.13E−2) +	3.20E−1 (5.15E−2) +	6.45E−1 (1.75E−2) +	7.58E−1 (4.80E−3)−	4.57E−1 (2.36E−2) +
	8	8.36E−1 (1.70E−3)	8.16E−1 (4.87E−3) +	8.13E−1 (4.73E−3) +	7.40E−1 (2.89E−2) +	8.13E−1 (4.66E−3) +	1.98E−1 (3.02E−2) +	6.53E−1 (4.73E−2) +	8.66E−1 (3.99E−3)−	4.02E−1 (2.25E−2) +
	10	9.16E−1 (1.77E−3)	9.07E−1 (3.42E−3) +	9.04E−1 (3.28E−3) +	7.86E−1 (4.85E−2) +	8.99E−1 (5.46E−3) +	2.26E−1 (6.94E−2) +	6.22E−1 (3.96E−2) +	9.19E−1 (3.53E−3)−	3.86E−1 (2.20E−2) +
WFG7	2	2.09E−1 (5.22E−4)	2.11E−1 (5.47E−3)≈	2.11E−1 (1.30E−3)−	1.94E−1 (3.66E−3) +	2.11E−1 (1.69E−3)−	2.19E−1 (1.14E−2)−	2.24E−1 (2.68E−2)−	2.10E−1 (8.99E−4)−	2.65E−1 (2.49E−2)−
	4	5.81E−1 (6.84E−4)	5.77E−1 (1.05E−3) +	5.71E−1 (1.61E−3) +	5.68E−1 (1.62E−3) +	5.67E−1 (2.00E−3) +	5.27E−1 (1.12E−2) +	5.38E−1 (5.29E−3) +	5.77E−1 (1.77E−3) +	4.31E−1 (1.88E−2) +
	6	7.47E−1 (1.55E−3)	7.31E−1 (6.44E−3) +	7.27E−1 (5.84E−3) +	7.12E−1 (7.43E−3) +	6.36E−1 (7.47E−2) +	3.80E−1 (3.50E−2) +	6.58E−1 (1.09E−2) +	7.57E−1 (3.25E−3)−	4.82E−1 (2.39E−2) +
	8	8.34E−1 (1.40E−3)	8.06E−1 (4.28E−3) +	8.01E−1 (3.91E−3) +	7.48E−1 (1.96E−2) +	7.88E−1 (6.86E−3) +	1.75E−1 (2.55E−2) +	6.91E−1 (2.48E−2) +	8.52E−1 (3.80E−3)−	4.41E−1 (2.10E−2) +
	10	9.14E−1 (1.54E−3)	8.99E−1 (2.55E−3) +	8.90E−1 (3.76E−3) +	8.31E−1 (9.93E−3) +	8.84E−1 (1.02E−2) +	1.81E−1 (5.05E−2) +	7.22E−1 (2.87E−2) +	9.00E−1 (3.79E−3) +	4.54E−1 (3.06E−2) +
WFG8	2	3.60E−1 (2.82E−2)	3.72E−1 (3.29E−2)≈	3.68E−1 (3.52E−2)≈	3.01E−1 (4.29E−2) +	3.72E−1 (3.20E−2)≈	3.51E−1 (4.78E−2)≈	3.06E−1 (3.88E−2) +	3.75E−1 (3.41E−2)−	3.28E−1 (6.52E−2) +
	4	5.28E−1 (2.41E−2)	5.08E−1 (2.47E−2) +	5.01E−1 (2.24E−2) +	5.09E−1 (2.39E−2) +	4.96E−1 (2.98E−2) +	4.84E−1 (1.86E−2) +	4.99E−1 (2.31E−2) +	5.35E−1 (2.99E−2)≈	3.74E−1 (3.81E−2) +
	6	7.09E−1 (1.24E−2)	6.82E−1 (2.93E−2) +	6.67E−1 (3.77E−2) +	6.15E−1 (3.22E−2) +	5.87E−1 (6.80E−2) +	2.89E−1 (4.70E−2) +	5.98E−1 (2.31E−2) +	7.37E−1 (6.72E−3)−	3.54E−1 (1.65E−2) +
	8	7.41E−1 (1.90E−2)	6.61E−1 (7.99E−3) +	6.65E−1 (1.65E−2) +	7.03E−1 (4.37E−2) +	6.64E−1 (1.49E−2) +	1.99E−1 (3.69E−2) +	5.98E−1 (3.91E−2) +	7.57E−1 (2.80E−2)−	3.61E−1 (3.96E−2) +
	10	8.49E−1 (3.26E−2)	8.20E−1 (2.46E−2) +	7.89E−1 (2.07E−2) +	7.20E−1 (7.11E−2) +	7.66E−1 (2.43E−2) +	1.77E−1 (3.98E−2) +	6.36E−1 (4.83E−2) +	8.57E−1 (4.25E−2)≈	3.52E−1 (3.24E−2) +
WFG9	2	2.31E−1 (4.57E−3)	2.30E−1 (3.93E−3)≈	2.32E−1 (5.25E−3)≈	2.19E−1 (3.02E−3) +	2.40E−1 (1.82E−2)−	2.70E−1 (2.71E−2)−	2.40E−1 (1.34E−2)−	2.38E−1 (1.59E−2)−	2.38E−1 (1.77E−2)−
	4	5.62E−1 (3.80E−3)	5.55E−1 (5.05E−3) +	5.58E−1 (2.97E−3) +	5.58E−1 (4.67E−3) +	5.51E−1 (6.71E−3) +	5.31E−1 (1.51E−2) +	5.36E−1 (8.54E−3) +	5.65E−1 (3.58E−3)−	4.82E−1 (1.61E−2) +
	6	6.86E−1 (7.73E−3)	6.55E−1 (1.33E−2) +	6.69E−1 (1.02E−2) +	6.63E−1 (1.27E−2) +	6.58E−1 (1.66E−2) +	3.80E−1 (3.66E−2) +	5.88E−1 (1.85E−2) +	7.01E−1 (7.46E−3)−	5.21E−1 (3.37E−2) +
	8	7.94E−1 (4.39E−3)	7.56E−1 (1.09E−2) +	7.64E−1 (8.73E−3) +	7.45E−1 (1.47E−2) +	7.55E−1 (1.78E−2) +	2.48E−1 (3.43E−2) +	6.33E−1 (2.83E−2) +	8.20E−1 (4.79E−3)−	5.53E−1 (2.59E−2) +
	10	8.68E−1 (4.44E−3)	8.48E−1 (7.61E−3) +	8.43E−1 (9.78E−3) +	7.84E−1 (1.73E−2) +	8.23E−1 (2.26E−2) +	2.14E−1 (3.06E−2) +	5.94E−1 (5.73E−2) +	8.48E−1 (7.46E−3) +	5.89E−1 (3.02E−2) +
		W–T–L	54–18–8	49–13–18	59–8–13	50–10–20	61–4–15	63–2–15	33–14–33	60–4–16

**Figure 3:**
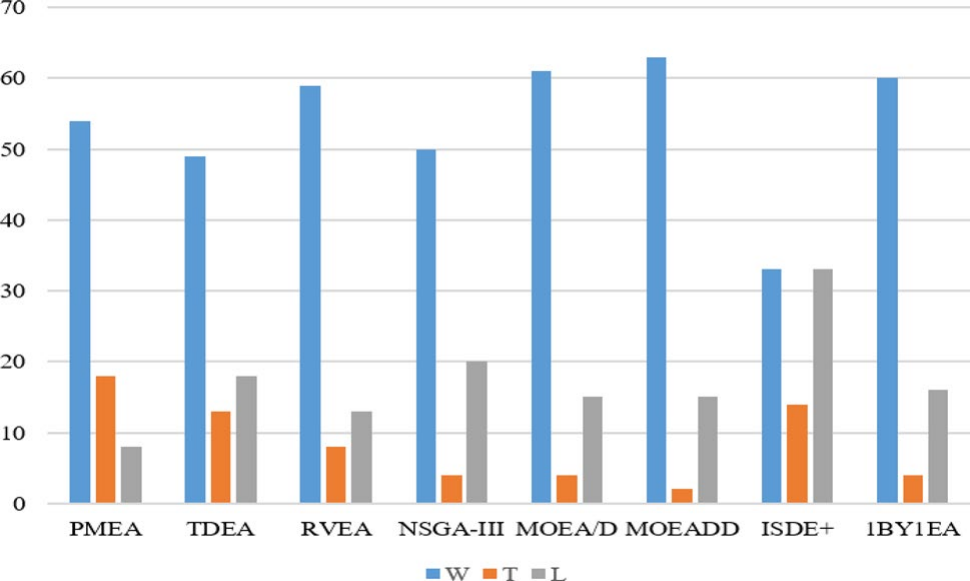
Performance comparison of HS-MOEA with the state-of-the-art algorithms.

As shown in Table 2 and Fig. 3, HS-MOEA significantly outperforms or comparable to PMEA, TDEA, RVEA, NSGAIII, MOEA/D, MOEA/DD, I_SDE_^+^ and ONEBYONE in 72/80 ≈ 90%, 62/80 ≈ 77.5%, 67/80 ≈ 83.75%, 60/80 ≈ 75%, 65/80 ≈ 81.25%, 65/80 ≈ 81.25%, 47/80 ≈ 58.75% and 64/80 ≈ 80% of cases, respectively. It can be observed that HS-MOEA consistently performs better or is similar to PMEA. Similarly, HS-MOEA outperforms ONEBYONE on most of the problems. However, in case of WFG1 and WFG2, ONEBYONE performs better over HS-MOEA. On the other hand; TDEA, RVEA and NSGA-III performs better than HS-MOEA on DTLZ1, WFG1 and WFG2 test problems. However, the improvements seems to be minimal compared to the advantages HS-MOEA achieves on other problems such as DTLZ5, DTLZ7, WFG3 ~ 9. MOEA/D and MOEA/DD seem to perform similarly compared to HS-MOEA, performing slightly better on DTLZ1. On WFG3, MOEA/D performs better than all the state-of-the-art algorithms, including HS-MOEA. However, the degraded performance of MOEA/D on the remaining 15 problems seems to outweigh the superior performance on WFG3.

Among the state-of-the-art algorithms, I_SDE_^+^ exhibits competitive performance compared to HS-MOEA. The superiority of I_SDE_^+^ compared to HS-MOEA can be seen on DTLZ1, WFG1, WFG6 and WFG9. The performance improvement is significant; however, HS-MOEA is also close. HS-MOEA has a slight advantage over I_SDE_^+^ on problems such as DTLZ7 and WFG2 that have disconnected PF.

To demonstrate the effectiveness of HS-MOEA, a more detailed analyse corresponding to DTLZ1 and DTLZ7 is presented. GD and Delta indicators that indicate the convergence and diversity performance of MOEAs are summarized in Tables 3 and 4, respectively. Lower values of both GD and Delta values indicate the superiority of the algorithm. The convergence (GD) of HS-MOEA is consistently better than PMEA. However, the convergence of HS -MOEA lags behind I_SDE_^+^ on DTLZ1, which was designed to test the convergence performance of MOEAs. However, the diversity (Delta) of HS -MOEA is consistently better than I_SDE_^+^. On the other hand, HS -MOEA fails to maintain the diversity with respect to PMEA on DLTZ7, which has discontinuous PF. In other words, the convergence of HS-MOEA is better or comparable to PMEA, while the diversity is better or comparable to I_SDE_^+^.

**Table 3:** Comparison of GD.

#	M	HS-MOEA	PMEA	ISDE +
DTLZ1	2	2.04E−4 (8.93E−6)	3.32E−2 (1.81E−1)≈	2.09E−2 (1.06E−1)≈
	4	2.70E−3 (2.86E−4)	1.46E−1 (2.21E−1) +	3.21E−3 (2.35E−3)≈
	6	4.44E−2 (9.67E−2)	6.21E−1 (5.09E−1) +	5.06E−3 (7.97E−5)−
	8	3.94E−2 (7.82E−2)	6.54E−1 (2.63E−1) +	5.34E−3 (7.63E−4)−
	10	1.93E−2 (4.21E−2)	8.00E−1 (2.67E−1) +	5.73E−3 (6.55E−5)-

**Table 4:** Comparison of delta.

#	M	HS-MOEA	PMEA	ISDE +
DTLZ7	2	4.47E−1 (3.16E−2)	2.00E−1 (2.75E−2)−	5.04E−1 (4.54E−2) +
	4	3.39E−1 (2.61E−2)	2.77E−1 (1.89E−2)−	3.33E−1 (2.63E−2)≈
	6	3.56E−1 (2.17E−2)	2.71E−1 (1.31E−2)−	3.94E−1 (4.47E−2) +
	8	2.90E−1 (3.42E−2)	2.20E−1 (1.23E−2)−	3.60E−1 (3.74E−2) +
	10	2.16E−1 (1.52E−2)	2.06E−1 (1.17E−2)−	3.15E−1 (3.78E−2) +

The improved performance of HS-MOEA is because it gets benefitted from both the best qualities of each component—(1) Pareto dominance’s ability to eliminate low-quality solutions, (2) Uniform weight vectors maintain the diversity, and (3) I_SDE_^+^ indicator enable both convergence and diversity in problems with MOPs with irregular or discontinuous PFs. Therefore, the performance of HS-MOEA is competitive or better in most cases. The significance is more visible in problems with discontinuous PFs such as DTLZ7.

**Figure 4:**
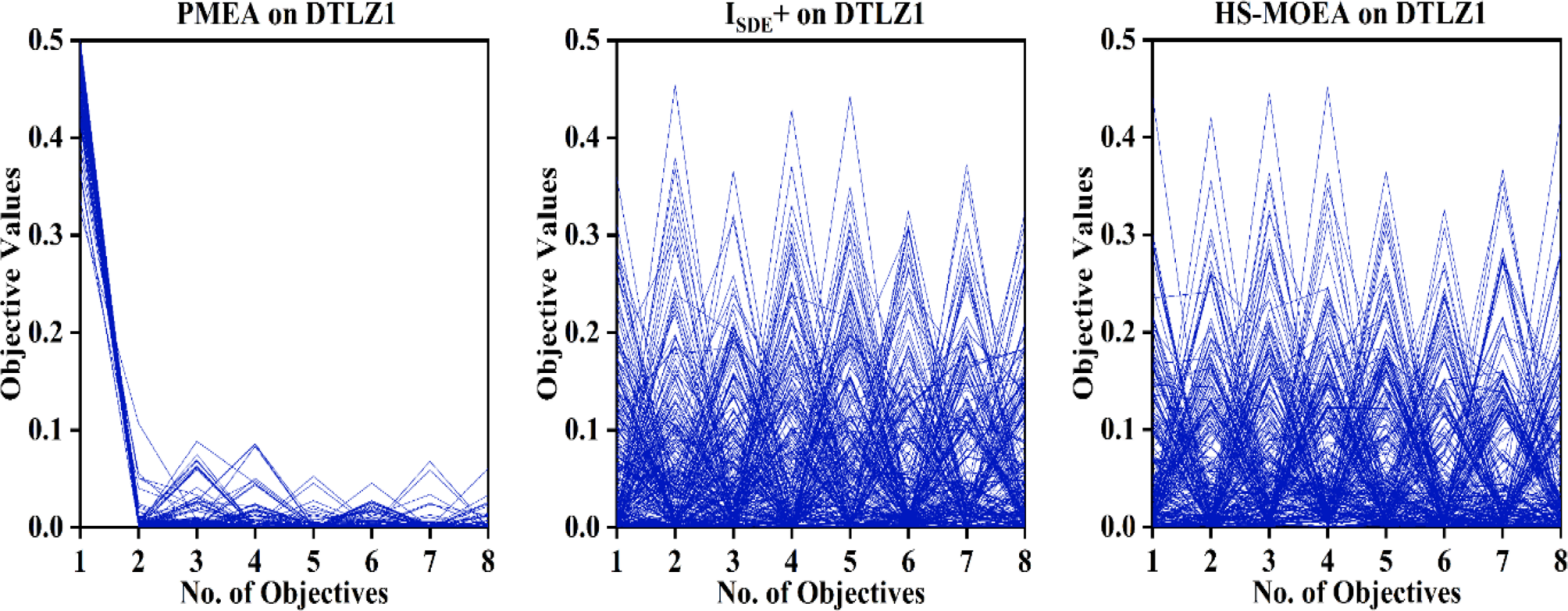
Parallel coordinates of the best solution set of PMEA, I_SDE_^+^ and HS-MOEA on 8-objective instance of DTLZ1.

**Figure 5:**
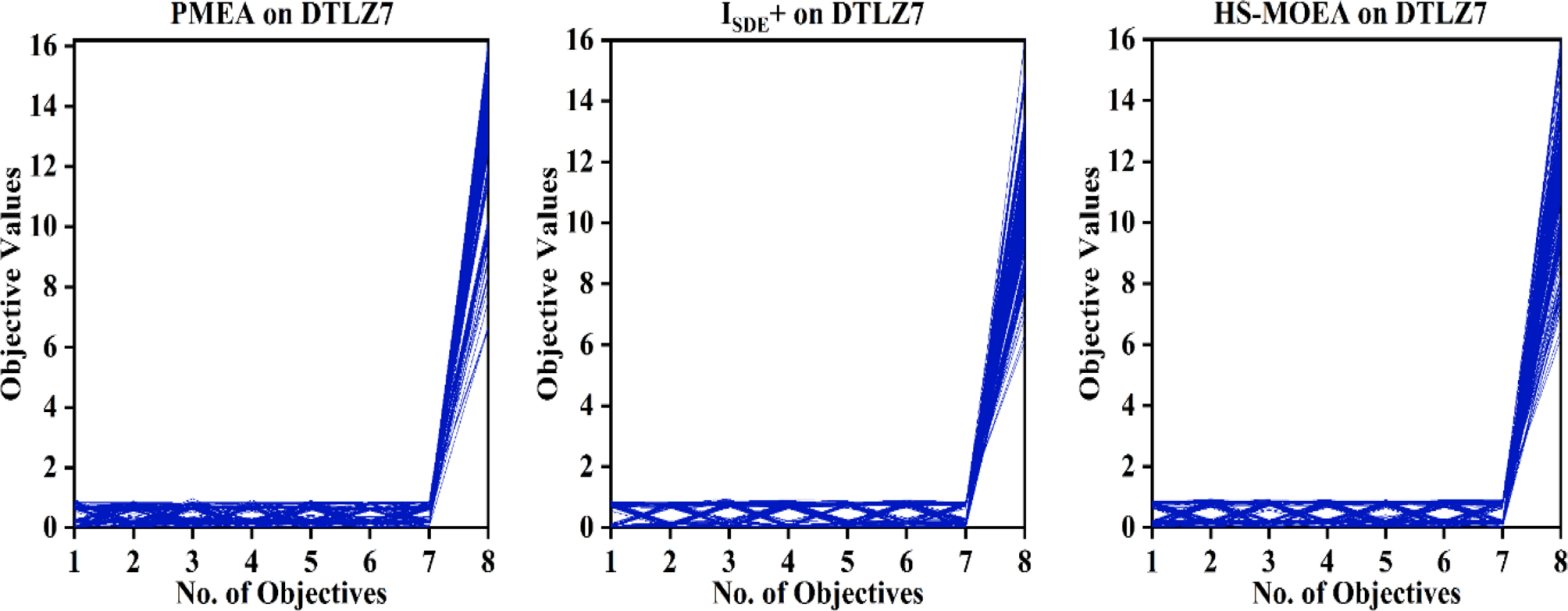
Parallel coordinates of the best solution set of PMEA, I_SDE_^+^ and HS-MOEA on 8-objective instance of DTLZ7.

Figures 4 and 5 present the parallel coordinates that describe the distribution of the solutions corresponding to PMEA, I_SDE_^+^ and HS-MOEA on 8-objective instances of DTLZ1 and DTLZ7. From the figures, it is evident that HS-MOEA is able to provide a well converged and diverse set of solutions compared to PMEA on both the DTLZ1 and DTLZ7 instances. However, the parallel coordinates of I_SDE_^+^ and proposed HS-MOEA seem nearly identical on both DTLZ1 and DTLZ7. From the results in Table 4, it is evident that I_SDE_^+^ slightly outperforms HS-MOEA on DTLZ1, which has continuous linear PF, while HS-MOEA performs better on DTLZ7 that has discontinuities in the PF.

## Conclusion

This paper proposes a Hybrid Selection based Multi/Many-objective optimization, named HS-MOEA. In HS-MOEA, a new environmental selection that benefits from the advantages of Pareto dominance, reference vectors and an indicator is proposed. HS-MOEA is compared with seven state-of-the-art MOEAs on a number of widely used test instances. Experimental results demonstrate the superiority of HS-MOEA among all compared algorithms, mainly on problems with discontinuous PFs such as DTLZ7. In the future, we would like to investigate the possibility of weight vector adaptation using the I_SDE_^+^ indicator. In other words, new positions of the ineffective weight vectors and the consequent adjustment of the effective weight vectors can be estimated by employing the indicator values.

## References

[1] Dutta, S. & Das, K. N. A survey on pareto-based eas to solve multi-objective optimization problems. *Soft Comput. Prob. Solving* 807–820. 10.1007/978-981-13-1595-4_64 (2019).

[2] Mashwani WK, Salhi A (2014). Multiobjective memetic algorithm based on decomposition. Appl. Soft Comput..

[3] Mashwani WK, Salhi A (2016). Multiobjective evolutionary algorithm based on multimethod with dynamic resources allocation. Appl. Soft Comput..

[4] Hisao, I., Noritaka, T. & Yusuke, N. in *2008 IEEE Congress on Evolutionary Computation (IEEE World Congress on Computational Intelligence).* 2419–2426.

[5] Zitzler, E., Laumanns, M. & Thiele, L. TIK-report 103 (2001).

[6] Deb K, Pratap A, Agarwal S, Meyarivan T (2002). A fast and elitist multiobjective genetic algorithm: NSGA-II. IEEE Trans. Evol. Comput..

[7] Laumanns M, Thiele L, Deb K, Zitzler E (2002). Combining convergence and diversity in evolutionary multiobjective optimization. Evol. Comput..

[8] Yuan Y, Xu H, Wang B, Yao X (2016). A new dominance relation-based evolutionary algorithm for many-objective optimization. IEEE Trans. Evol. Comput..

[9] Dutta, S., M, S. S., Mallipeddi, R., Das, K. N. & Lee, D.-G. A Mating Selection Based on Modified Strengthened Dominance Relation for NSGA-III. *Mathematics* **9**. doi:10.3390/math9222837 (2021).

[10] Purshouse RC, Fleming PJ (2007). On the evolutionary optimization of many conflicting objectives. IEEE Trans. Evol. Comput..

[11] Deb K, Jain H (2014). An evolutionary many-objective optimization algorithm using reference-point-based nondominated sorting approach, part i: Solving problems with box constraints. IEEE Trans. Evol. Comput..

[12] Zitzler, E. & Künzli, S. in *International conference on parallel problem solving from nature.* 832–842 (Springer).

[13] Pamulapati T, Mallipeddi R, Suganthan PN (2019). I_SDE_+—An indicator for multi and many-objective optimization. IEEE Trans. Evol. Comput..

[14] Li B, Tang K, Li J, Yao X (2016). Stochastic ranking algorithm for many-objective optimization based on multiple indicators. IEEE Trans. Evol. Comput..

[15] Bader J, Zitzler E (2011). HypE: An algorithm for fast hypervolume-based many-objective optimization. Evol. Comput..

[16] Zhang Q, Li H (2007). MOEA/D: A multiobjective evolutionary algorithm based on decomposition. IEEE Trans. Evol. Comput..

[17] Zhang, Q., Liu, W. & Li, H. in *2009 IEEE Congress on Evolutionary Computation.* 203–208.

[18] Das I, Dennis JE (1998). Normal-Boundary Intersection: A New Method for Generating the Pareto Surface in Nonlinear Multicriteria Optimization Problems. SIAM J. on Optimization.

[19] Ishibuchi, H., Doi, K., Masuda, H. & Nojima, Y. in *2015 IEEE Symposium Series on Computational Intelligence.* 861–868.

[20] Jain H, Deb K (2014). An evolutionary many-objective optimization algorithm using reference-point based nondominated sorting approach, part ii: Handling constraints and extending to an adaptive approach. IEEE Trans. Evol. Comput..

[21] Cheng R, Jin Y, Olhofer M, Sendhoff B (2016). A reference vector guided evolutionary algorithm for many-objective optimization. IEEE Trans. Evol. Comput..

[22] Li K, Deb K, Zhang Q, Kwong S (2015). An evolutionary many-objective optimization algorithm based on dominance and decomposition. IEEE Trans. Evol. Comput..

[23] Xu H, Zeng W, Zeng X, Yen GG (2021). A polar-metric-based evolutionary algorithm. IEEE Trans. Cybern..

[24] Liu, H., Chen, L., Zhang, Q. & Deb, K. in *2016 IEEE Congress on Evolutionary Computation (CEC).* 4763–4769.

[25] Asafuddoula M, Singh HK, Ray T (2018). An enhanced decomposition-based evolutionary algorithm with adaptive reference vectors. IEEE Trans. Cybern..

[26] Cai X, Mei Z, Fan Z (2018). A decomposition-based many-objective evolutionary algorithm with two types of adjustments for direction vectors. IEEE Trans. Cybern..

[27] Liu, Q., Jin, Y., Heiderich, M. & Rodemann, T. in *2019 IEEE Congress on Evolutionary Computation (CEC).* 1726–1733.

[28] Ishibuchi H, Setoguchi Y, Masuda H, Nojima Y (2017). Performance of decomposition-based many-objective algorithms strongly depends on pareto front shapes. IEEE Trans. Evol. Comput..

[29] Deb, K., Thiele, L., Laumanns, M. & Zitzler, E. in *Proceedings of the 2002 Congress on Evolutionary Computation. CEC'02 (Cat. No.02TH8600).* 825–830 vol.821.

[30] Huband S, Hingston P, Barone L, While L (2006). A review of multiobjective test problems and a scalable test problem toolkit. IEEE Trans. Evol. Comput..

[31] Liu Y, Gong D, Sun J, Jin Y (2017). A many-objective evolutionary algorithm using a one-by-one selection strategy. IEEE Trans. Cybern..

